# Evaluating the Effect of Glass and Carbon Fiber Mesh on 3D-Printed Concrete Performance

**DOI:** 10.3390/ma19081639

**Published:** 2026-04-20

**Authors:** Emad Janghorban, Arpan Joshi, Florindo José Mendes Gaspar

**Affiliations:** Centre for Rapid and Sustainable Product Development (CDRSP), Polytechnic Institute of Leiria, 2430-028 Marinha Grande, Portugal; arpan.joshi@ipleiria.pt (A.J.); florindo.gaspar@ipleiria.pt (F.J.M.G.)

**Keywords:** 3D-printed concrete, concrete reinforcement, carbon fiber mesh, glass fiber mesh, flexural strength, interlayer bonding, additive manufacturing

## Abstract

**Highlights:**

- ARMO-mesh 500/500 doubles flexural strength (up to a 100% increase).
- Glass fiber mesh yields only marginal gains (~3–12%).
- Larger beam-like specimens show a clear size effect (reduced strength).
- Wide porous carbon meshes enable automated reinforcement.
- Apparent superior interlayer bonding and post-cracking toughness are achieved.
- Corrosion resistance and sustainability benefits over steel are observed.

**Abstract:**

Additive manufacturing of concrete offers reduced waste, faster construction, and design freedom, yet effective reinforcement integration remains a major challenge due to weak interlayer bonding and anisotropy. Most prior studies focus on vertical reinforcement, short fibers, or metallic systems, achieving modest flexural improvements (15–60%). This study evaluates horizontal continuous reinforcement using glass fiber mesh and two carbon fiber meshes (ARMO-mesh 200/200 and 500/500) integrated during 3D printing. The methods include extrusion-based printing of small (four-layer) and beam-like (eight-layer) specimens, both printed and cast, followed by three-point flexural and compression tests at 7 and 28 days under vertical and horizontal loading. The results show that ARMO-mesh 500/500 significantly enhances flexural strength—up to 100% over unreinforced controls (e.g., 24.4 kNm vs. 12.2 kNm in small specimens at 28 days) and ~60% over ARMO-mesh 200/200, while glass mesh provides only marginal gains (~12%). Carbon meshes also improve post-cracking toughness and apparent interlayer cohesion. A pronounced size effect reduces nominal strength in larger specimens. These findings demonstrate that wide-format porous carbon meshes offer a scalable, corrosion-resistant solution for load-bearing 3D-printed concrete elements, advancing automated digital construction.

## 1. Introduction

Concrete is the most widely used structural material worldwide; however, conventional construction methods face significant challenges, including high costs, substantial material waste (largely from disposable formwork), and considerable energy consumption [1,2]. The advent of 3D printing (additive manufacturing) in construction promises transformative benefits, such as up to a 50% reduction in construction time, 30–60% material savings, and 50–80% faster processes when extrusion-based techniques are scaled [3]. Comprehensive reviews underscore its potential to lower CO_2_ emissions, minimize onsite waste, and enable complex, formwork-free geometries that are often uneconomical with traditional approaches [4,5]. Real-world examples, including the Tecla House (Italy), the Milestone Project (The Netherlands), and initiatives in Dubai, demonstrate substantial reductions in cost, time, labor, and resources [6,7,8].

Despite these advantages, a critical barrier to broad adoption of 3D-printed concrete is the effective integration of reinforcement. Conventional strategies—such as pre-installed steel meshes, mesh–mold systems, or post-print rebar insertion—frequently compromise geometric freedom, disrupt full automation, or demand significant manual intervention [9,10,11]. Recent reviews consistently identify weak interlayer bonding, mechanical anisotropy, and inadequate reinforcement—particularly under flexural loading—as primary limitations confining most printed elements to non-structural or low-load applications [12,13,14]. Adhesion at the interlayer interface is the fundamental phenomenon governing the mechanical performance of 3D-printed concrete [15]. Reinforcement has thus emerged as a priority research area, encompassing manual rebar cages, post-tensioning, in-process cable/mesh deposition, U-shaped nails, and short fibers [16,17,18].

Current efforts emphasize automated in-process reinforcement, including steel bars, short fibers, and mesh systems [19,20]. For instance, in-process U-nail insertion has achieved ~145% increase in interlayer tensile strength and ~220% in shear strength through mechanical interlocking [18]. Mesh-based approaches, such as overlapping steel meshes via specialized nozzles, have markedly improved flexural capacity and ductility [21]. However, most studies prioritize vertical or isotropic reinforcement with metallic systems; a notable gap persists in continuous horizontal reinforcement using advanced non-metallic meshes (e.g., carbon fiber) aligned with principal tensile stresses and fully compatible with automated printing [22,23]. While fiber reinforcement (carbon, basalt, glass, polymers) enhances ductility, tensile performance, and interlayer adhesion, systematic investigations of wide-format carbon fiber meshes—particularly assessing geometry, thickness, porosity, and surface morphology impacts on flexural behavior in printed mortars—remain limited [23,24,25].

Prior work with short fibers or slender carbon elements typically yields modest flexural gains (15–30%) [13], while textile or glass-fiber meshes as stay-in-place forms improve load capacity by ~60%, primarily via ductility rather than strength doubling [14]. Emerging cable- and mesh-reinforced systems show larger flexural and post-cracking improvements when continuously bonded along print paths, yet these remain predominantly steel-based and lack optimization for lightweight, corrosion-resistant use or detailed quantification of mesh properties [16,20,24].

This study addresses these gaps by quantitatively evaluating horizontal integration of glass fiber mesh and two carbon fiber mesh grades (ARMO-mesh 200/200 and 500/500) in 3D-printed mortar. The continuous, wide-format carbon mesh—embedded during printing—is hypothesized to provide superior mechanical interlocking and stress transfer due to its geometry and porosity. Unreinforced controls, glass-reinforced specimens, and carbon-reinforced specimens (printed and cast, varying sizes) were compared via three-point flexural and compression tests. The results demonstrate that ARMO-mesh 500/500 enhances flexural strength by up to 100% over controls, substantially exceeding typical gains from fibers or glass meshes while markedly improving interlayer cohesion and post-cracking toughness. These findings establish a practical benchmark for automated reinforcement in digital concrete construction, with implications for scalability, sustainability, and next-generation structural elements.

## 2. Materials and Methods

The following sections detail the materials selected for this study as well as the methods employed for specimen preparation and testing.

### 2.1. Materials

This section provides a detailed description of all materials used in the preparation of mortar mixtures and reinforced specimens.

#### 2.1.1. Mortar Materials

The mortar mixture was designed for extrusion-based 3D printing, ensuring pumpability, buildability, and rapid setting. The primary binder was Ordinary Portland Cement (OPC) CEM I 42.5 R supplied by Secil (Lisboa, Portugal), with Blaine fineness 350–400 m^2^/kg, density 3.15 g/cm^3^, and initial setting time 90–120 min. The aggregate was natural siliceous sand supplied by Imosa, Lda. (Guia-Pombal, Portugal) with maximum particle size 500 µm. To optimize packing and workability, 10% of the sand (by mass) was replaced with high-purity limestone filler (>97% CaCO_3_, D50 = 6.8 µm). A polycarboxylate-based superplasticizer (Woerment FM 422) supplied by Sika AG (Baar, Switzerland) was added at 1% of cement weight to adjust viscosity and prevent segregation. Tap water was used, with water/binder ratio 0.30.

**Figure 1:**
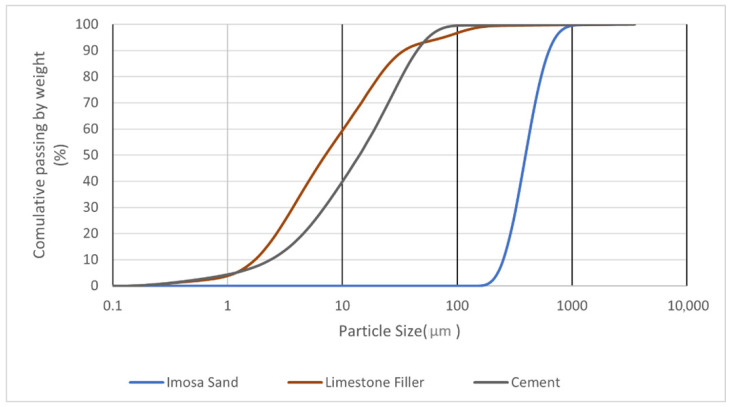
Particle size distribution.

Particle size distributions (D10, D50, D90) are summarized in Table 1 and illustrated in Figure 1.

#### 2.1.2. Reinforcing Materials

**Figure 2:**
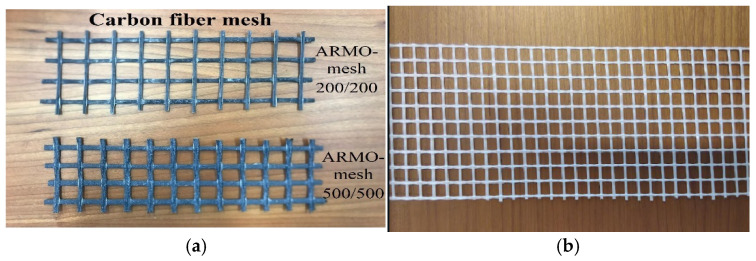
(**a**) Carbon fiber mesh; (**b**) glass fiber mesh.

Various reinforcement meshes were selected based on a market survey of commercially available products. Specifically, a glass fiber mesh supplied by Würth Portugal, Lda. (Sintra, Portugal) and two types of carbon fiber mesh (ARMO-mesh 200/200 and 500/500) supplied by S&P Clever Reinforcement Ibérica, Lda. (Amora, Portugal) were used. These meshes are illustrated in Figure 2. The choice of these mesh products reflects a balance between availability, cost-effectiveness, mechanical performance, and integration ease with 3D printing. ARMO-mesh 500/500 was selected for its superior mechanical enhancement, providing maximum structural benefit but at a relatively higher cost, making it suitable for critical load-bearing applications. ARMO-mesh 200/200 offers a compromise between performance and cost, while glass fiber mesh represents an accessible, low-cost alternative with moderate mechanical contribution, acting as a practical benchmark for comparison. This range covers actual engineering trade-offs encountered in construction practice.

The selection of ARMO-mesh 500/500 (thicker, more porous) was driven by its superior capacity to bridge cracks and transfer tensile stresses aligned with principal flexural directions in 3D-printed elements. ARMO-mesh 200/200 offers a cost-performance balance, while glass fiber mesh serves as a low-cost benchmark. This range directly addresses the structural needs of horizontal reinforcement in printed concrete.

To ensure clarity and transparency, the technical specifications for the glass fiber mesh and both types of carbon fiber mesh are provided in separate tables (Table 2 and Table 3), as reported directly by the manufacturers. All values in Table 2 and Table 3 are reported exactly as provided in the manufacturers’ technical datasheets to ensure transparency and reproducibility.

**Figure 3:**
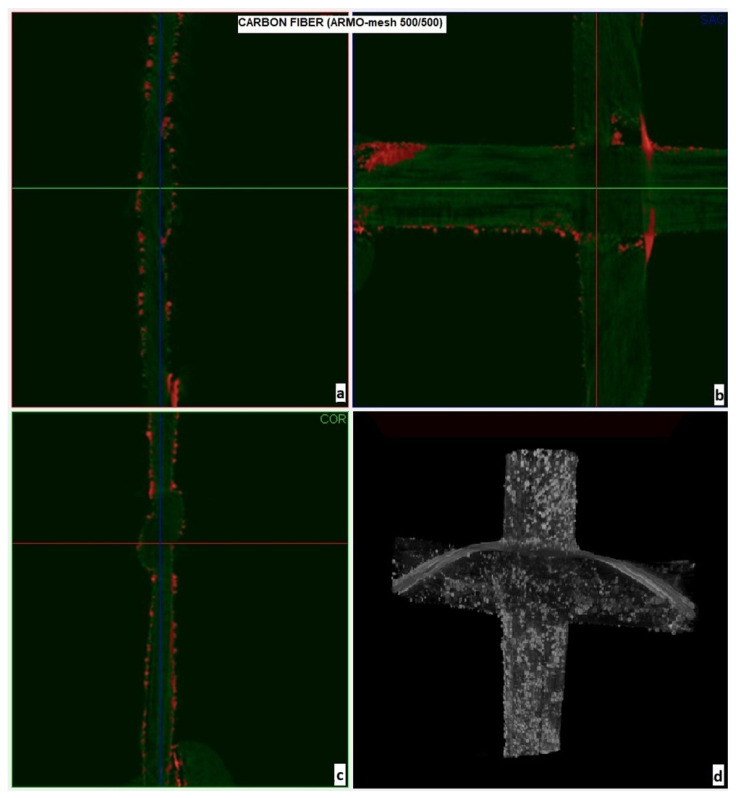
Micro-CT images of ARMO-mesh 500/500: (**a**,**c**) side view of the mesh structure; (**b**,**d**) front view showing the distribution of mesh openings and surface roughness (red points = internal pores, white regions = rough surface).

**Figure 4:**
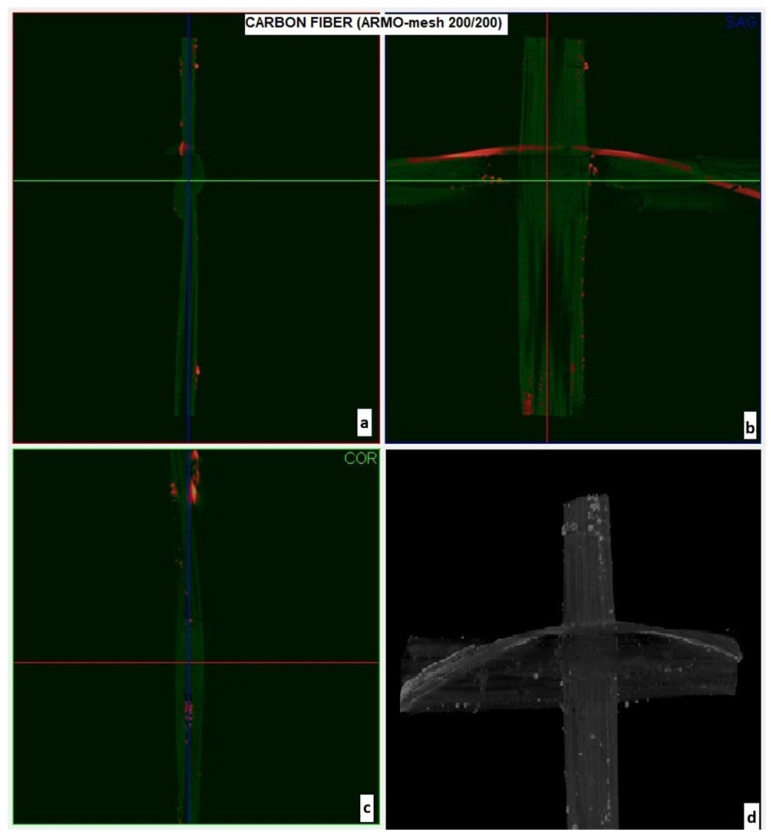
Micro-CT images of ARMO-mesh 200/200: (**a**,**c**) side views displaying strand continuity and internal density; (**b**,**d**) front views revealing mesh uniformity, minimal internal pores (red points), and a smooth surface profile.

The porosity and surface morphology of the carbon meshes were further examined using Micro-CT imaging (Figure 3 and Figure 4). In these X-ray micro-computed tomography images, white/bright regions correspond to the carbon fibers (higher X-ray attenuation due to greater material density), grey regions represent any residual mortar matrix or background material, and dark/black areas indicate voids and pores within the mesh structure. ARMO-mesh 500/500 exhibited higher porosity and thickness, promoting better interlayer interlocking, while ARMO-mesh 200/200 was denser and smoother.

Note that, in Table 2, all listed properties are directly provided in the manufacturer’s data sheet. Tensile strength in N/5 cm represents the maximum load measured per 5 cm strip, as defined by Würth.

Note that, in Table 3, all technical data are as provided in the official product documentation by S&P Clever Reinforcement Company.

As summarized in Table 2 and Table 3, all key mechanical and physical properties are reported exactly as specified by the mesh manufacturers. For parameters such as “Tensile strength (N/5 cm)” used in the glass fiber product, the manufacturer’s test setup and reporting format are retained to ensure transparency and data consistency. Specifications not directly comparable (such as MPa for carbon meshes versus N/5 cm for glass mesh) are clearly indicated. This format ensures that no manufacturer information is altered or recalculated and that all details provided can be appropriately referenced for future research or replication.

Micro-CT imaging was used to visualize porosity and surface morphology of the carbon meshes (Figure 3 and Figure 4). ARMO-mesh 500/500 exhibited higher porosity and thickness, promoting better interlocking, while ARMO-mesh 200/200 was denser and smoother. Quantitative surface analysis (e.g., roughness metrics) is planned for future work.

Figure 3b,d display the front view of the ARMO-mesh 500/500. These images emphasize the spatial arrangement of the mesh’s openings and the distribution of surface pores. Red points indicate internal cavities throughout the mesh structure, while white regions (particularly in Figure 3d) highlight areas of increased surface roughness and porosity. This dual perspective—side and front—enables a comprehensive understanding of both the three-dimensional architecture and the pore structure, which are critical for mechanical performance and bonding within the printed concrete.

To further investigate the geometric and surface properties of ARMO-mesh 200/200, micro-CT imaging was performed. Figure 4 presents the detailed micro-CT results for this mesh. In Figure 4a,c, the side views allow for an assessment of the continuity, strand thickness, and internal compactness of the mesh structure, aiding in a direct comparison with the properties observed for ARMO-mesh 500/500.

Figure 4b,d illustrate the front view of ARMO-mesh 200/200, capturing the uniformity of mesh openings and its notably smooth surface morphology. In these projections, the limited occurrence of red indicators reveals that internal porosity and void content are significantly lower than in ARMO-mesh 500/500. The absence of extensive white regions further confirms reduced surface roughness. This combination of observations highlights the finer, more compact, and less porous nature of ARMO-mesh 200/200. In correlation with the data presented in Table 3, ARMO-mesh 500/500 not only exhibits higher porosity but also greater thickness, resulting in a softer and more flexible texture compared to the denser and smoother ARMO-mesh 200/200. Such structural differences are expected to distinctly influence the mesh’s mechanical behavior and bonding performance in reinforced concrete applications.

While Figure 3 and Figure 4 provide useful visualizations of mesh geometry, pore distribution, and approximate roughness, the interpretation of surface properties in this work is primarily qualitative. The micro-CT imaging enabled visual differentiation between denser and more porous mesh types; however, quantitative measurements of surface properties—such as porosity percentage, roughness mean value, fiber orientation, and strand diameter distribution—were not directly performed. In future work, detailed surface characterization using 3D profilometry, image analysis, and quantitative porosity metrics will be pursued to provide an objective basis for the observed differences.

The mechanical performance and overall behavior of fiber-reinforced concrete systems depend strongly on the intrinsic properties of the mesh fibers. In this study, ARMO-mesh 200/200 and 500/500 are manufactured with high-strength, corrosion-resistant carbon fibers, while the glass fiber mesh is based on alkali-resistant (AR) glass. Carbon fibers are characterized by their exceptionally high tensile strength (typically above 3000 MPa), high elastic modulus, chemical inertness, and complete immunity to corrosion, making them especially suitable for aggressive environments and long-term applications. Their low weight and high flexibility further facilitate handling and installation, especially in automated or complex geometries.

In contrast, glass fiber mesh offers a lower tensile strength and modulus but provides good chemical stability due to its alkali-resistant composition. Nevertheless, glass fibers may suffer from long-term degradation in highly alkaline environments unless carefully protected by the surrounding matrix.

Both mesh types are produced with proprietary coatings (such as amorphous silica for S&P meshes) to enhance the bond with concrete and mortar. The mesh geometry—including strand diameter, opening size, and mesh thickness—directly influences the degree of mechanical interlocking, crack-bridging capability, and the efficiency of stress transfer between the reinforcement and the cementitious matrix. In sum, carbon fiber meshes deliver superior mechanical performance and durability, whereas glass fiber meshes may be considered a cost-effective alternative for situations with moderate mechanical demands and less severe environmental exposure.

#### 2.1.3. Mortar Composition

In this composition, aggregate and cement were combined in a ratio of 2:1. A total of 10% of the sand mass (by weight) was replaced with high-purity limestone filler (see Table 4 and Table 5 for exact batch proportions). In addition, a superplasticizer (Woerment FM 422) was added in an amount of 1% of the cement weight. The water/binder ratio was 0.30 in the composition, and tap water was used for mixing. The approximate batch proportions are shown in Table 5, with all component weights rounded upwards for clarity and practicality. The total weight per batch is approximately 72 kg.

The mixture proportions correspond to approximately 350 kg/m^3^ cement, 700 kg/m^3^ sand, 70 kg/m^3^ limestone filler, 262.5 kg/m^3^ water, and 3.5 kg/m^3^ superplasticizer (water/binder = 0.30).

The proportioning, mixing, and material selection for the mortar composition were carried out in accordance with the European Standard EN 196-1 [26], which specifies procedures for preparing standard cement mortars for mechanical strength testing. All constituent materials—cement, aggregate, limestone filler, water, and superplasticizer—were measured and combined as prescribed by this standard to ensure consistency and comparability of results. The mortar composition and mixing were performed according to EN 196-1 [26]. In addition, the mechanical properties of the mortar (including compressive strength of cast specimens) were evaluated using the same standard EN 196-1 [26] to ensure consistency and direct comparability of results across all tests. To demonstrate the effectiveness of the reinforcement and allow for a meaningful comparison of results, the same concrete composition and material conditions were used in all tests.

The parameters of the manufactured samples were as follows. All samples used the same mortar composition (Table 4), with meshes placed horizontally between layers. At least 9 samples per configuration were produced for statistical reliability. Determined indicators were as follows: flexural strength (FS, MPa) via three-point flexural strength (Equations (1) and (2)), compressive strength (CS, MPa) for unreinforced cast samples (Equation (1)), and qualitative observations of failure modes, ductility, and interlayer bonding. Results were processed as averages with standard deviations, compared across variables to quantify reinforcement effects.

### 2.2. Preparation of the Samples

The following subsections outline the specific procedures used for the preparation, printing, and handling of all specimens evaluated in this study.

#### 2.2.1. Printing of the Specimens

**Figure 5:**
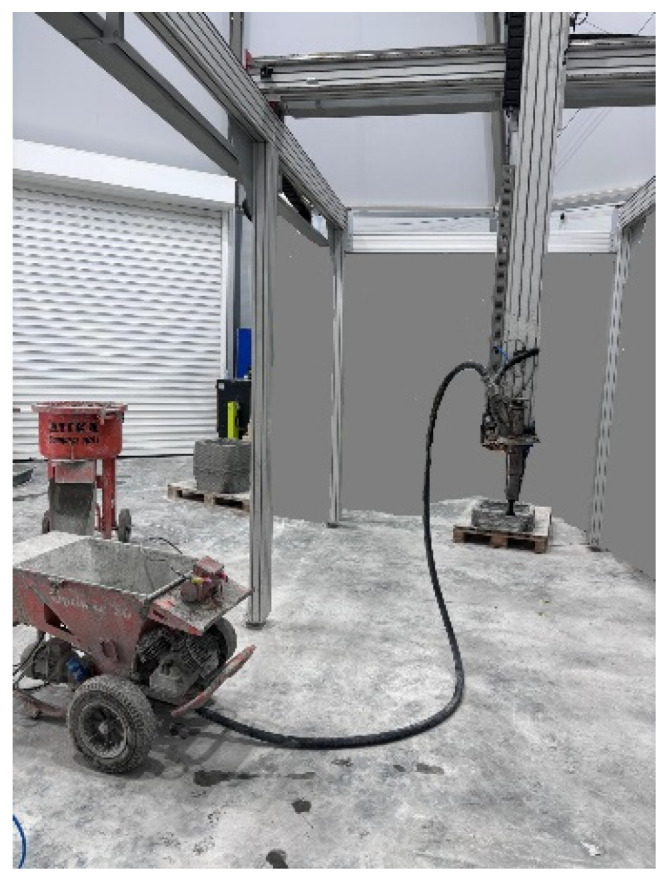
Printing equipment.

All specimens were fabricated using a custom-designed Cartesian 3D printer (see Figure 5), adapted specifically for cementitious materials. The mortar was mixed in an Atika Compact Screed Mixer (100 L capacity) supplied by ALTRAD LESCHA ATIKA GmbH (Burgau, Germany) to ensure homogeneity. After mixing, the mortar was allowed to rest for 15 min before printing to achieve the desired consistency for extrusion.

**Figure 6:**
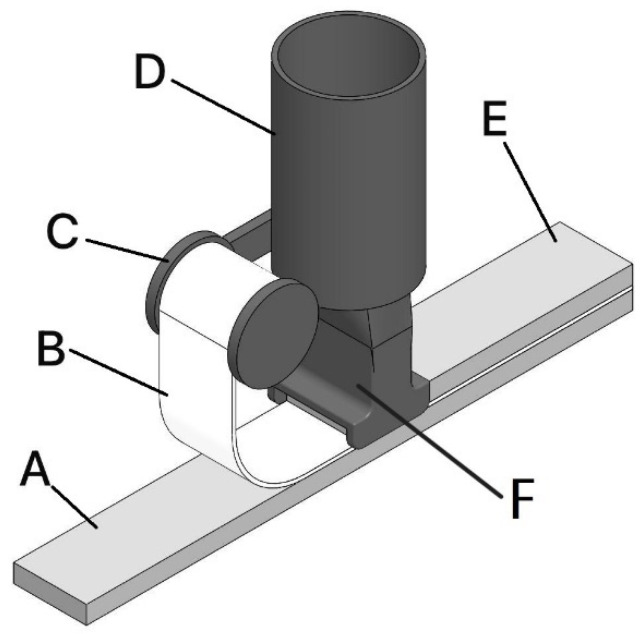
Concept of the print head. A: First printing layer; B: Mesh reinforcement; C: Spool; D: Extrusion head; E: Second printing layer; F: Printing head.

During the printing process, mortar was extruded layer by layer through a specially designed print head (concept illustrated in Figure 6). For each sample, the print path and dimensions were programmed in advance, with linear specimens printed in four layers (10 mm each) and beam-like specimens produced in eight layers to reach a total thickness of 16 cm.

**Figure 7:**
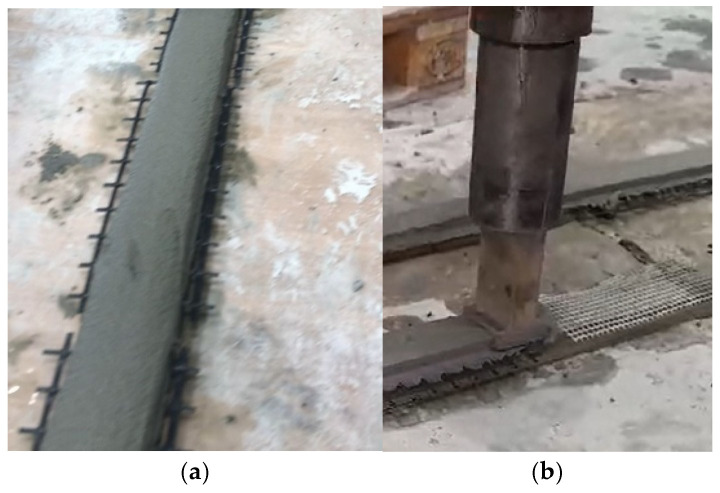
3D-printing test; (**a**) carbon fiber mesh; (**b**) glass fiber mesh.

All printed layers were uniformly controlled to a thickness of 10 mm. The minor color variation observed in some specimens (Figure 7) is attributed to slight differences in surface moisture and drying rate during layer-by-layer deposition and has no effect on the mechanical performance or interlayer bonding.

The incorporation of reinforcement meshes (ARMO-mesh 200/200, ARMO-mesh 500/500, and glass fiber mesh) was performed manually in this study. Meshes were pre-moistened to enhance adhesion and placed horizontally at designated layers during printing. For linear (smaller) samples, the mesh was positioned only between the first and second printed layer, whereas in beam-like specimens, a moistened mesh was inserted at every layer. This procedure is clarified schematically in Figure 6 and illustrated in practice in Figure 7a (carbon fiber mesh) and Figure 7b (glass fiber mesh). The mesh was only placed on the first layer for smaller samples, after which the remaining layers were printed. However, for beam-like specimens, moistened meshes were incorporated into all printed layers. Additionally, unreinforced control samples were fabricated alongside the reinforced ones as a baseline for comparison.

It should be noted that, although manual placement was used for greater experimental control and adjustment at this research scale, mechanical or fully automated mesh placement is feasible and widely studied for industrial-scale 3D printing. Automated systems can improve placement precision and repeatability; however, they may introduce technical and cost challenges, especially for complex designs. Future work is planned to directly compare fully automated and manual mesh placement methods, evaluating differences in build efficiency, structural integration, and scale-up feasibility in real construction scenarios.

Throughout the printing process, the print head precisely shaped each mortar layer to the target geometry, ensuring consistent thickness and coverage over the mesh. Unreinforced specimens were also printed under the same conditions to serve as control samples for direct comparison.

Once printing was complete and the mortar had reached initial set (approximately 30 min), all printed specimens were cut using a metal blade into lengths of 20 cm (for linear specimens) or 60 cm (for beam-like specimens) to facilitate curing and subsequent testing. Cast specimens were demolded after 7 days. At least nine samples were produced for each type of reinforcement and the control group.

Environmental conditions in the laboratory were maintained at 20 ± 2 °C and 50 ± 5% relative humidity during fabrication and setting. All sample preparation, handling, and documentation adhered to relevant best practices for 3D-printed mortar research.

#### 2.2.2. Cast Specimens Preparation

To further investigate and ensure the quality control of the printed samples, casting samples were produced similarly to the printed ones. This approach considers that, in casting samples, the process of shaking the mold helps release trapped air bubbles, resulting in better bonding between the reinforcement and the concrete as well as improved compaction.

**Figure 8:**
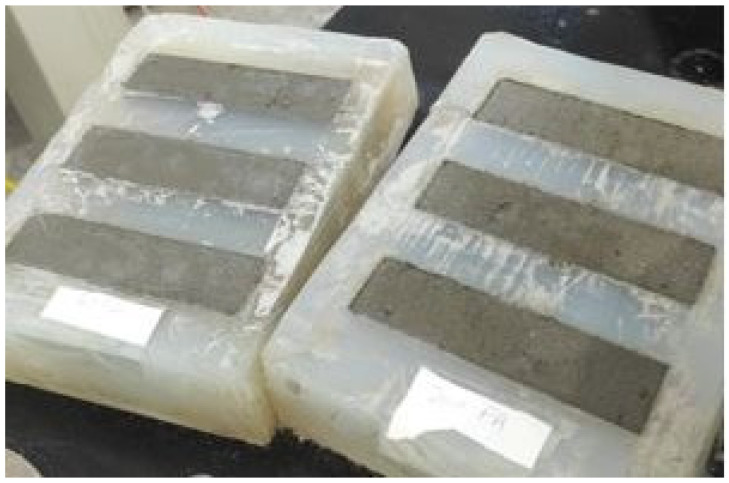
Casted test specimens.

To prepare the casted specimens, molds were used, having dimensions of 40 mm × 40 mm × 160 mm. The mold was first filled with 10 mm-thickness mortar, corresponding to the first layer of the printed specimens, and immediately after, the moistened reinforcement was placed. Then, the mold was filled with the mortar. To ensure proper compacity of the mortar, the molds were carefully shaken (Figure 8).

### 2.3. Experimental Program

The experimental program systematically evaluated the influence of reinforcement type on the mechanical performance of 3D-printed and cast concrete. The following parameters were varied:

- Reinforcement type: None (control), glass fiber mesh, ARMO-mesh 200/200, ARMO-mesh 500/500.
- Fabrication method: 3D printing vs. casting.
- Specimen size: Small (40 mm × 40 mm × 160 mm, 4 layers) vs. beam-like (approximately 100 mm × 160 mm × 600 mm, 8 layers).
- Curing age: 7 days and 28 days.
- Loading direction: Vertical (perpendicular to print layers) and horizontal (parallel to layers).

Adhesion and interlayer bond performance were evaluated indirectly through three-point bending tests by examining post-cracking load retention, displacement tolerance (up to 10 mm without mesh rupture), and overall flexural behavior. Direct bond strength measurements (e.g., pull-out or shear tests) were not conducted in this study. All specimens used the same mortar composition, with meshes placed horizontally between layers and pre-moistened for better adhesion. At least nine specimens were manufactured per configuration to ensure statistical reliability. Determined indicators included flexural strength (FS, MPa) via three-point flexural strength, compressive strength (CS, MPa) for unreinforced cast samples, and qualitative observations of failure modes, ductility, and interlayer bonding. Results were processed as averages with standard deviations.

All mechanical test procedures were strictly conducted in accordance with international standards to ensure data reliability and reproducibility. Three-point flexural tests were performed in accordance with EN 12390-5 [27], while compressive strength tests followed EN 196-1 [26]. All mechanical properties were evaluated using a calibrated universal testing machine (100 kN capacity) in compliance with EN ISO 7500-1 [28]. Below, step-by-step details for specimen curing, preparation, and testing are described. All testing equipment, including the universal testing machine (capacity: 100 kN), was calibrated prior to experimentation according to the manufacturer’s specifications and international standards (EN ISO 7500-1 [28]). The machine’s force measurement system was verified for linearity and repeatability, with maximum error kept within ±1% of applied load, as specified for research-grade instruments.

#### 2.3.1. Small Printed Samples and Cast Samples

**Figure 9:**
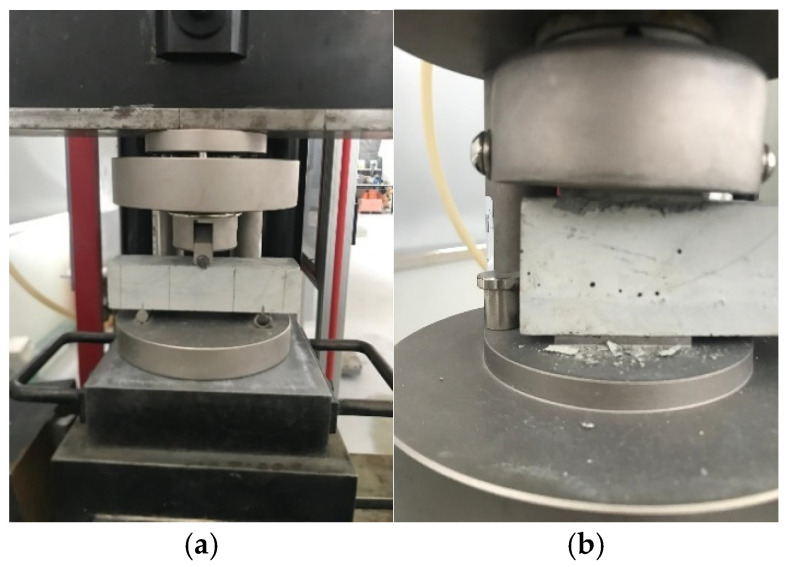
Mechanical testing machines; (**a**) flexural strength test; (**b**) compression test.

All printed and cast specimens were cured under identical laboratory conditions to ensure reliable comparison of mechanical properties. The samples were kept for 7 and 28 days in a climate-controlled room maintained at 20 °C and 50% relative humidity, shielded from direct sunlight and air drafts. This setup minimized environmental variations and enhanced reproducibility by preventing premature drying, surface cracking, and other factors that might influence the hydration and hardening process. Throughout the curing period, specimens remained undisturbed on stable shelves to avoid mechanical stress and ensure consistent quality. These curing conditions were strictly applied to all printed and cast samples in this study, enabling an accurate assessment of mechanical performance across different reinforcement types and fabrication methods. After 7 days of curing and then 28 days later, the casted samples were removed from the mold, and the printed samples were cut to specific dimensions. Care was taken to ensure that all samples were uniformly cut to the same size: 40 mm in width, 40 mm in height, and 160 mm in length. Three-point flexural strength, conducted according to EN 12390-5 [27], and compression tests for cast samples (EN 196-1 [26]) were performed using a testing machine with a capacity of 100 kN, as shown in Figure 9.

**Figure 10:**
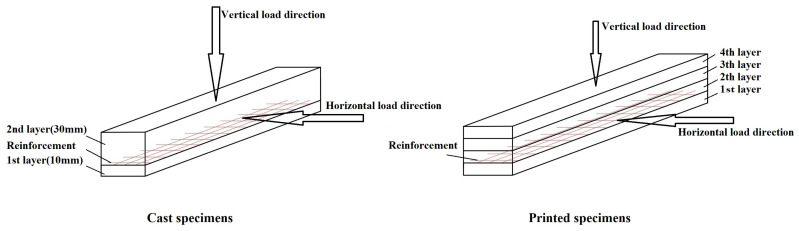
Load direction of cast and printed specimens.

To evaluate the effects of mesh reinforcement on 3D-printed specimens, both printed and cast specimens containing each reinforcement layout were subjected to flexural strength with both vertical and horizontal loading directions (see Figure 10). For the flexural strength measurement, specimens (printed and cast) were loaded centrally at a constant rate of 50 N/s in a three-point flexural strength, specifically between two individual 100 mm-wide floor supports (Figure 9a), until failure. For beam-like specimens, a span of 480 mm and a loading rate of 90 N/s were used. The supports and loading roller were aligned as per the standard protocol, and the loading process was recorded by the automatic data acquisition system of the instrument.

In all cases, reinforcements were placed horizontally between layers during printing to specifically target interlayer bond strength. To ensure uniformity and reproducibility, the surfaces at the interlayer interfaces were carefully prepared, and the mesh reinforcements were pre-moistened before placement to enhance bonding with the concrete matrix.

After the flexural strength, for casted samples without reinforcement, the two broken parts were positioned on the test setup with an area of 40 mm × 40 mm (Figure 9b) to measure the compressive strength at a loading force of 400 N/s.

For each configuration and reinforcement type, at least three replicates were tested, and the maximum applied load and failure mode were automatically recorded by the testing device. The flexural strength for each specimen type (with or without reinforcement) was calculated as the average of three specimens. Similarly, for casted samples, the compressive strength of samples without reinforcement was determined as the average of the six specimens from the flexural strength tests.

This combined approach enabled reproducible and reliable measurement of both overall flexural performance and the specific contribution of horizontal reinforcement to interlayer bond strength (see Figure 9 and Figure 10). The load data recorded by the testing machine were used to calculate the flexural strength and compressive strength of the specimens according to Equations (1) and (2), respectively.

CS = F/A(1)

where CS is the compression strength (MPa), F is the compression load at failure, and A is the cross-section area in which the load is being applied (40 mm × 40 mm).

(2) $$FS=\frac{3FL}{2BH^{2}}$$

where *F*S is the flexural strength or modulus of rupture (MPa), F is the load at failure (N), L is the span of the simple supports (100 mm), *B* is the width of the specimen (mm), and H is the thickness of the specimen (mm).

#### 2.3.2. Beam-like Specimens

**Figure 11:**
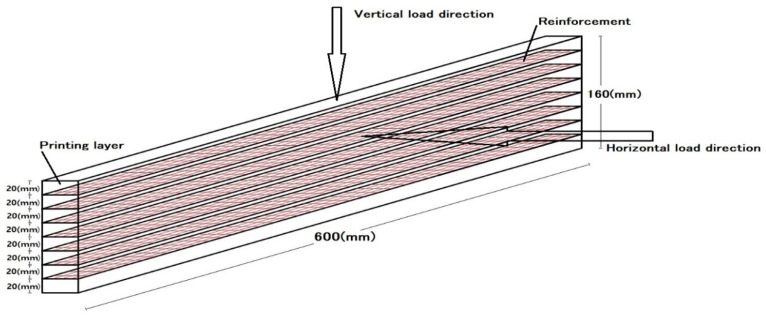
Schematic representation of the beam-like specimen (8 layers, approximate dimensions: 100 mm width × 160 mm height × 600 mm length). Note: The schematic is not drawn to exact scale. Actual specimen width is 100 mm, as stated in the text (Section 2.3).

For beam-like specimens (600 mm in length and 160 mm in height), three-point flexural tests were conducted by EN 12390-5 [27] standards, like the smaller printed samples. These tests were performed 28 days after printing using a testing machine with a capacity of 100 kN. To further evaluate the impact of mesh reinforcement on 3D-printed concrete, printed samples with each type of reinforcement were subjected to flexural tests under both vertical and horizontal loading directions (see Figure 11).

**Figure 12:**
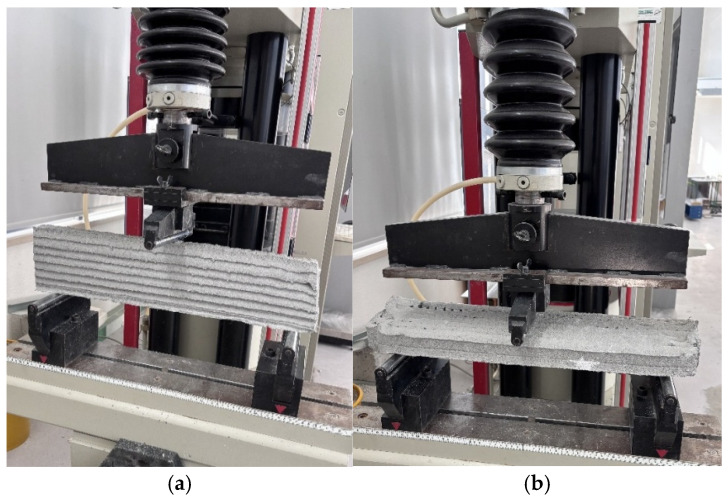
Flexural tests: (**a**) Vertical load direction; (**b**) Horizontal load direction. Slight color variation over height in (**b**) is due to differences in surface moisture and drying rate between consecutively printed layers and does not indicate any variation in layer thickness. All layers were uniformly controlled to 10 mm thickness.

To measure the flexural strength, the samples were loaded vertically and horizontally at a force rate of 90 N/s during a three-point flexural test. The test was 350 at the center of the sample, positioned between two separate supports spaced 480 mm apart (Figure 12), until failure occurred. The maximum loads were automatically recorded by the testing machine. The flexural strength for each type of sample (with or without reinforcement) was determined as the average of three specimens. The load data recorded by the testing machine was used to calculate the flexural strength of the samples according to Equation (2), where FS is the flexural strength or modulus of rupture (MPa), F is the load at failure (N), L is the span of the simple supports (480 mm), B is the width of the specimen (mm), and H is the thickness of the specimen (mm).

The careful implementation of these standardized test protocols allowed for accurate comparison of the mechanical performance and reinforcement effect for all sample types.

**Figure 13:**
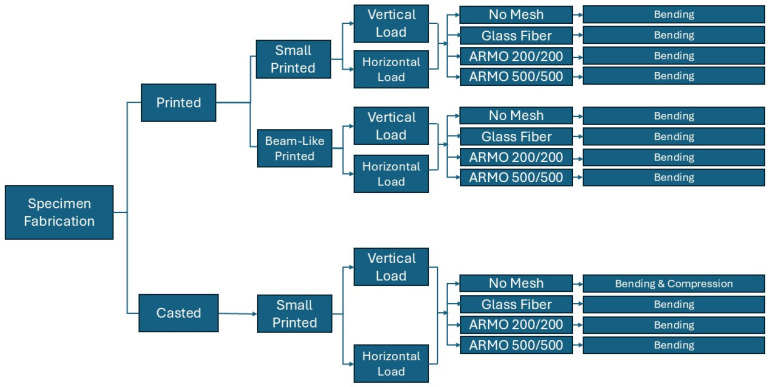
Workflow diagram showing the selection, preparation, and testing process for all sample types.

To clearly summarize the types and numbers of samples, preparation steps, and loading procedures, two elements are presented. The experimental workflow—including material selection, reinforcement type, fabrication (3D printing or casting), curing, cutting, and loading method—is visualized in a flowchart (Figure 13).

The detailed quantity and grouping of each sample, along with their reinforcement configuration and specific loading type, are listed in Table 6.

## 3. Results

The following subsections present the experimental outcomes for all sample series studied. All results are reported as mean values ± standard deviation. In all bar charts, the height of each rectangle corresponds to the mean value, and the error bars represent the standard deviation (n ≥ 9 per configuration).

### 3.1. Printed Specimens

This section presents the mechanical test results for printed and cast specimens, including flexural strength under vertical and horizontal loading, compressive strength of unreinforced cast samples, and observations of failure behavior. All results are reported as mean values ± standard deviation.

#### 3.1.1. Vertical Load Direction in Printed Specimens 

While the laboratory conditions provide optimal conditions for observing the highest effectiveness of the reinforcement, the real-world samples reflect the practical application of the reinforcement. In these practical scenarios, various factors such as ambient air temperature, pressure duration, and pump pressure can affect the strength of the concrete. However, this study aimed to ensure that all conditions were kept consistent.

##### Small Samples (Vertical Load)

The average results of the flexural tests on small samples for horizontal reinforcement with different types of reinforcement under vertical loading after 7 and 28 days of printing are shown in Table 7. All results reported in Table 7, Table 8, Table 9, Table 10, Table 11, Table 12, Table 13 and Table 14 represent the calculated compressive strength (CS) or flexural strength (FS) of the tested specimens, expressed in MPa. These values were obtained using the maximum load recorded during testing and the corresponding equations provided in Section 2.3.

**Figure 14:**
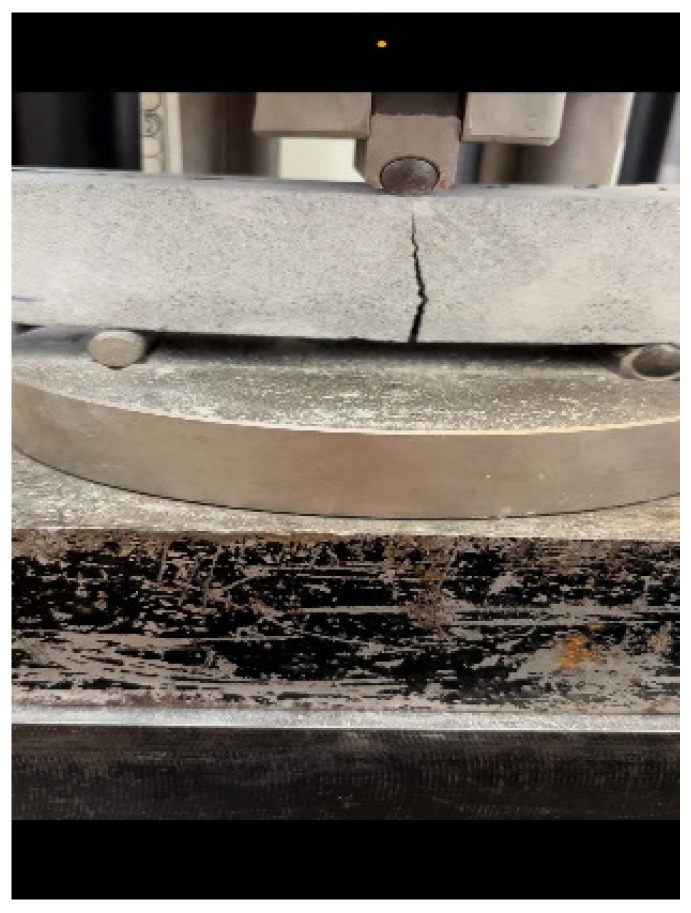
Flexural strength on ARMO-mesh 500/500 (carbon fiber samples).

In the three-point flexural tests on 7-day cured small specimens, samples reinforced with ARMO-mesh 500/500 sustained the highest maximum loads. As the applied load increased, cracks formed in the mortar matrix, and loading continued until the machine reached its maximum displacement of 10 mm, at which point the test was manually stopped (Figure 14). The ARMO-mesh 500/500 remained intact without rupture throughout the test.

Samples reinforced with glass fiber mesh showed maximum loads similar to those of unreinforced specimens. In these samples, the mesh ruptured simultaneously with the mortar matrix upon cracking.

Samples reinforced with ARMO-mesh 200/200 exhibited maximum loads higher than those of unreinforced specimens but lower than those reinforced with ARMO-mesh 500/500.

In 28-day cured small specimens, the highest maximum loads were recorded for samples reinforced with ARMO-mesh 500/500, followed by those reinforced with ARMO-mesh 200/200. Samples reinforced with glass fiber mesh displayed maximum loads comparable to those of unreinforced specimens.

**Figure 15:**
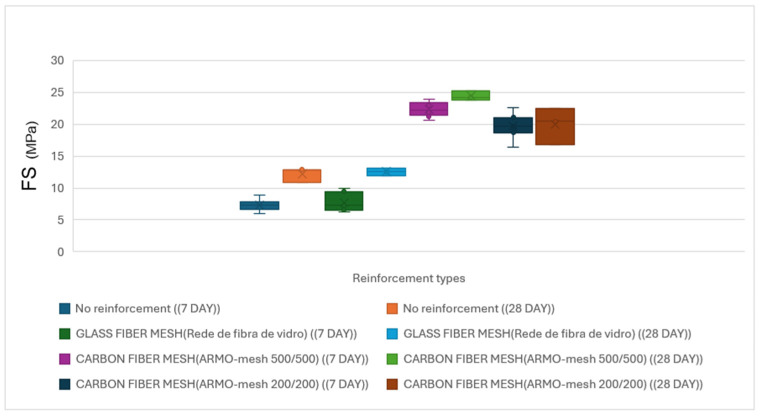
Average flexural strength of small printed specimens under vertical loading at 7 and 28 days. The height of each bar represents the mean value; error bars indicate standard deviation (n ≥ 9 per configuration).

A comparison of 7-day and 28-day results showed an increase in maximum load for unreinforced specimens and those reinforced with glass fiber mesh. In contrast, maximum loads in specimens reinforced with carbon fiber meshes (both ARMO-mesh 200/200 and 500/500) showed little change between 7 and 28 days (Figure 15).

**Figure 16:**
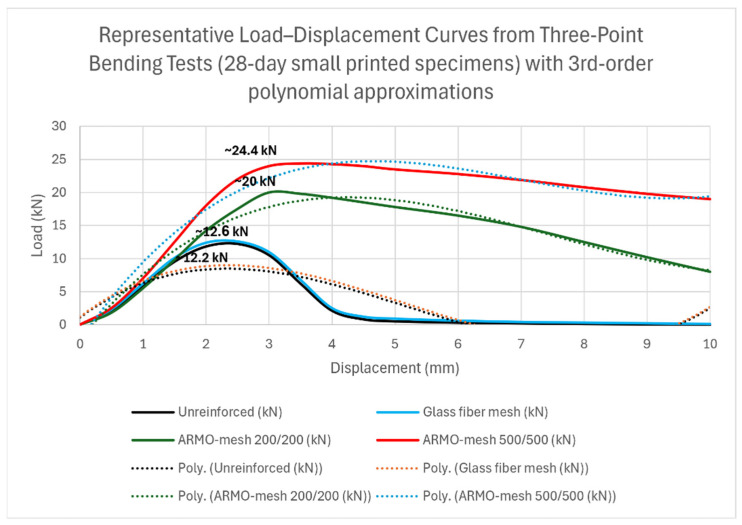
Representative load–displacement curves from three-point flexural tests on 28-day small printed specimens. Solid lines show experimental data; dotted lines represent 3rd-order polynomial approximations fitted to the post-peak region. The ARMO-mesh 500/500 specimen maintains significant load-carrying capacity up to the machine displacement limit of 10 mm without rupture, demonstrating superior ductility and energy absorption compared to unreinforced and glass mesh specimens.

Representative load–displacement curves from the three-point flexural tests are presented in Figure 16. These curves were constructed directly from measured load and crosshead displacement data and show the complete response up to the machine displacement limit of 10 mm. Third-order polynomial approximation lines (dotted) were fitted exclusively to the post-peak region to highlight the non-linear behavior, ductility differences, and sustained load-carrying capacity of the reinforced specimens.

##### Beam-like Specimens (Vertical Load)

The average results of three-point flexural tests performed on beam-like specimens after 28 days of curing are presented below.

Due to the size and geometry of the beam-like specimens, compressive strength tests were not conducted. Only flexural tests were carried out. All tests on beam-like specimens were limited to samples cured for 28 days. Glass fiber mesh reinforcement was excluded from the beam-like specimen tests.

Flexural strength results under vertical loading at 28 days are shown in Table 8.

#### 3.1.2. Horizontal Load Direction in Beam-like Specimens

The horizontal loading investigations are subdivided for clarity into separate analyses of small and large specimens, each discussed in detail in the following subsections.

##### Small Samples (Horizontal Load)

Table 9 shows the findings from the flexural tests conducted on small printing specimens that were reinforced with various types of reinforcement under horizontal loading.

**Figure 17:**
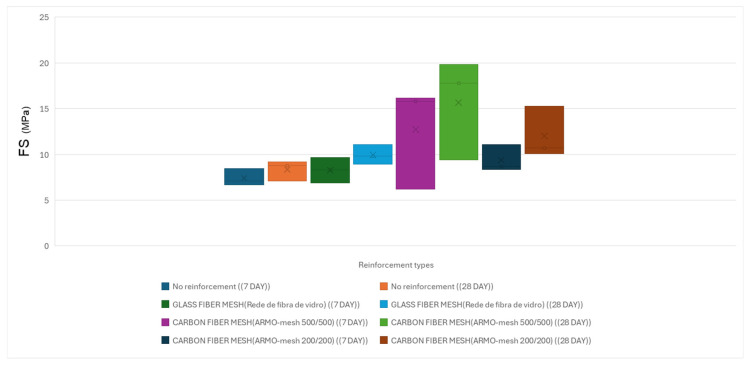
Flexural strength of printed specimens under horizontal loading at 7 and 28 days.

Representative load–displacement or stress–strain curves for small specimens under horizontal loading are shown in Figure 17.

##### Beam-like Specimens (Horizontal Load)

The average results of three-point flexural strength under horizontal loading on beam-like specimens cured for 28 days are presented below.

Tests were performed on 28-day cured specimens reinforced with ARMO-mesh 200/200 and ARMO-mesh 500/500. The flexural strength results under horizontal loading are shown in Table 10.

### 3.2. Cast Specimens

Cast specimens were tested under both vertical and horizontal loading configurations. The results are presented in the following subsections.

#### 3.2.1. Vertical Load Direction in Cast Specimens

The average results of three-point flexural strength under vertical loading on 7-day and 28-day cast specimens are presented in Table 11.

**Figure 18:**
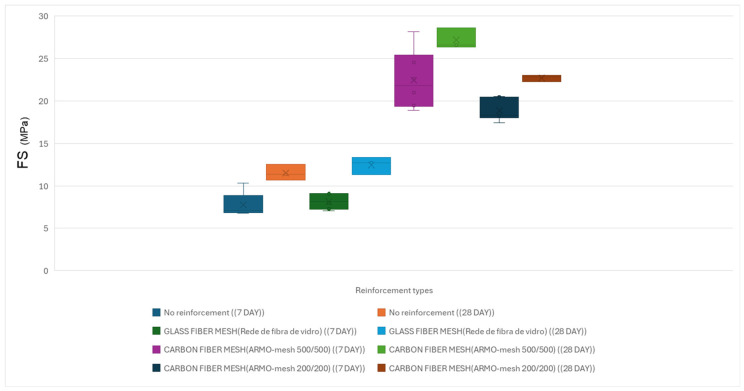
Average flexural strength of beam-like specimens under horizontal loading at 7 and 28 days. The height of each bar represents the mean value; error bars indicate standard deviation (n ≥ 9 per configuration).

Flexural strength results under vertical loading are illustrated in Figure 18.

The average compressive strength results for unreinforced cast specimens under vertical loading are presented in Table 12.

#### 3.2.2. Horizontal Load Direction in Cast Specimens

Table 13 summarizes the results of flexural and compressive strength tests under horizontal loading on 7-day and 28-day cast specimens reinforced with various types of reinforcement.

**Figure 19:**
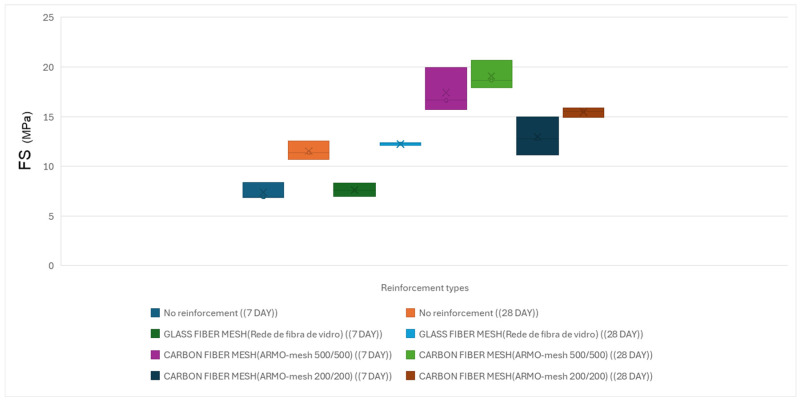
Flexural strength of cast specimens under horizontal loading at 7 and 28 days.

Flexural strength results under horizontal loading are illustrated in Figure 19.

The average compressive strength results for unreinforced cast specimens under horizontal loading are presented in Table 14.

Representative stress–strain diagrams for cast specimens under flexural strength (vertical and horizontal loading) are included in Figure 16 (refer to Section Small Samples (Vertical Load) for details on diagram construction and normalization).

### 3.3. Failure Modes

Failure modes were strongly dependent on both the reinforcement type and the specimen geometry. Unreinforced and glass fiber mesh-reinforced specimens (both small and beam-like) exhibited classic brittle failure under both vertical and horizontal loading: a single dominant vertical crack formed at mid-span, propagated through multiple printed layers, and resulted in immediate and complete loss of load-carrying capacity with no post-peak resistance.

In contrast, specimens reinforced with ARMO-mesh 200/200 and especially ARMO-mesh 500/500 showed pronounced ductile behavior. The continuous, wide-format carbon fiber mesh effectively bridged the crack, remained intact without rupture, and sustained significant post-peak load up to 10 mm mid-span deflection. This crack-bridging mechanism was observed consistently in both small specimens (160 mm length, four layers) and beam-like specimens (600 mm length, eight layers) under vertical and horizontal loading directions. The superior performance of ARMO-mesh 500/500 compared to ARMO-mesh 200/200 is attributed to its greater thickness and higher porosity, which enhanced mechanical interlocking with the surrounding mortar.

**Figure 20:**
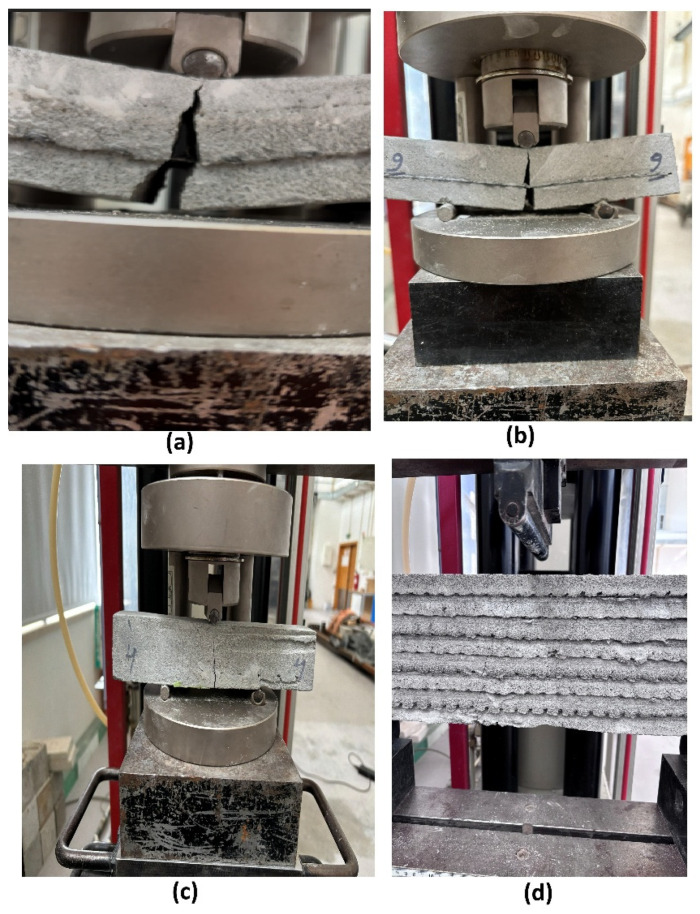
Representative failure modes after three-point bending tests for different reinforcement types and specimen geometries: (**a**) Ductile crack-bridging by carbon fiber mesh (ARMO-mesh 500/500, small specimen, vertical loading)—the mesh remains intact and holds the specimen together after concrete cracking; (**b**) Top view showing post-peak integrity (small specimen, ARMO-mesh 500/500); (**c**) Side view during/after loading (specimen 4, ARMO-mesh 200/200, small specimen); (**d**) Layered crack propagation through multiple printed layers (beam-like specimen, ARMO-mesh 500/500). Note: Panels (**a**–**c**) show small printed specimens (160 mm length, 4 layers); panel (**d**) shows a beam-like specimen (600 mm length, 8 layers). All panels illustrate ductile behavior of carbon mesh-reinforced specimens; brittle failure of unreinforced and glass mesh specimens is described in the text above.

These differences demonstrate the superior energy absorption, ductility, and apparent interlayer cohesion provided by the horizontal integration of wide-format carbon fiber meshes (Figure 20).

## 4. Discussion

The results of this study demonstrate that horizontal integration of continuous carbon fiber meshes during extrusion-based 3D printing substantially enhances the flexural strength and apparent interlayer cohesion in printed mortar specimens. In particular, ARMO-mesh 500/500 produced the greatest increase in flexural strength, reaching up to 100% higher maximum loads compared to unreinforced controls in small specimens and up to 70% higher than glass fiber mesh-reinforced specimens. ARMO-mesh 200/200 provided intermediate improvements (approximately 60%), while glass fiber mesh yielded only marginal gains (3–12%).

These enhancements are primarily attributed to the continuous, wide-format geometry of the carbon meshes, which enables efficient stress transfer across printed layers and effective crack bridging after initial cracking. The pronounced post-peak load retention and sustained load-carrying capacity beyond peak load in carbon mesh-reinforced specimens indicate improved ductility and energy absorption compared to the brittle failure observed in unreinforced and glass mesh-reinforced samples. The superior performance of ARMO-mesh 500/500 relative to ARMO-mesh 200/200 is likely linked to its greater thickness (0.105 mm vs. 0.044 mm) and higher porosity, which facilitate better mechanical interlocking and reduce potential slip at the fiber–mortar interface.

Although direct bond tests (e.g., shear or pull-out) were not conducted in this study, the marked increases in ultimate load, post-peak load-bearing capacity, sustained displacement tolerance up to 10 mm without mesh rupture, and superior post-cracking behavior with carbon meshes provide strong indirect evidence of apparent interlayer cohesion and mechanical interlocking. This is consistent with the role of continuous horizontal reinforcement in bridging weak interlayer planes and promoting more uniform stress distribution across layers. Future work will include dedicated pull-out tests to directly quantify bond strength.

A clear size effect was observed in the flexural results: beam-like specimens (600 mm length, eight layers) exhibited substantially lower nominal flexural strength compared to small specimens (160 mm length, four layers). This reduction is consistent with Bažant’s Size Effect Law for quasi-brittle materials [19] but is also influenced by the different reinforcement ratios between the two specimen sizes (mesh placed in every layer in beam-like specimens versus only one layer in small specimens). In such layered systems, the effect is likely amplified by inherent anisotropy and interlayer weaknesses, underscoring the need for scale-aware design when translating laboratory results to structural elements.

Compared to prior studies achieving only 15–60% flexural gains with short random fibers, glass textile meshes or vertical/isotropic metallic systems [12,13,14,15,16,17,18], the present approach with horizontal ARMO-mesh 500/500 delivers up to 100% strength improvement together with markedly superior post-cracking toughness. This advancement stems from the wide-format porous structure, optimal alignment with principal tensile stresses, and fully automated in-process integration—features that overcome the limitations of manual placement, narrow strips, and corrosion-prone steel reinforcements used previously. Moreover, the carbon meshes provide inherent corrosion resistance and sustainability benefits over steel while enabling scalable digital construction without compromising geometric freedom. Table 15 summarizes key comparisons with selected studies, highlighting the novelty of this method for load-bearing 3D-printed elements.

Although direct bond tests were not conducted in this study, the superior post-cracking behavior and higher ultimate loads with carbon meshes strongly indicate improved interlayer cohesion and mechanical interlocking. Future work will include dedicated pull-out tests to quantify bond strength.

Overall, this study demonstrates that mesh geometry (thickness, porosity, width), placement direction (horizontal), and material (carbon vs. glass) are decisive factors in overcoming anisotropy and weak interlayer bonding in 3D-printed concrete. The results establish a new performance benchmark for automated, corrosion-resistant reinforcement in digital construction.

## 5. Limitations

The manual placement of meshes limits immediate scalability to fully automated processes; potential risks include delamination in humid environments if bonding is insufficient. Key limitations include the absence of direct interlayer bond tests (shear, pull-out), relatively small specimen sizes that may not fully represent large-scale structural behavior, and the higher cost of carbon meshes compared to glass alternatives.

## 6. Conclusions

Horizontal integration of wide-format carbon fiber meshes during 3D printing significantly improves the flexural performance of concrete elements. ARMO-mesh 500/500 consistently provided the highest enhancement in maximum load capacity (up to 100% over unreinforced controls), followed by ARMO-mesh 200/200, while glass fiber mesh yielded only marginal gains. The superior result with carbon meshes are linked to their continuous structure, thickness, porosity, and horizontal placement, leading to better stress transfer, crack bridging, and apparent interlayer cohesion (inferred from flexural and post-cracking performance, see Figure 20), as evidenced by increased ultimate loads, post-peak load retention, and displacement tolerance.

A pronounced size effect was observed, with beam-like specimens showing lower nominal flexural strength than small specimens, consistent with the Size Effect Law for quasi-brittle materials. This highlights the importance of scale considerations in design and testing of 3D-printed concrete.

The approach offers practical advantages for engineering applications, enabling reliable load-bearing capacity in non-critical to moderately loaded elements (e.g., walls, beams, complex geometries) while supporting sustainability through reduced material waste, elimination of formwork, and lower CO_2_ emissions. Compared to conventional reinforcement methods (short fibers, glass textiles, U-nails, steel meshes), continuous carbon mesh integration achieves substantially higher flexural gains with corrosion resistance and lightweight characteristics.

The present study focused on flexural and compressive performance as the primary loading modes relevant to load-bearing 3D-printed concrete elements. Direct tensile tests and comprehensive durability assessments (e.g., corrosion resistance, freeze–thaw cycling, or long-term alkaline exposure) were, therefore, not included in the current experimental program but are planned for future work to provide a more comprehensive evaluation of the reinforced systems.

Future work should prioritize:

- Development of automated systems for simultaneous horizontal and vertical mesh placement during printing.
- Direct interlayer bond testing (shear, pull-out) and multi-axial loading experiments.
- Long-term durability assessments under environmental exposure.
- Exploration of hybrid reinforcements and scaling to full-scale structural prototypes and field trials.

These findings establish continuous carbon fiber mesh reinforcement as a promising strategy for improving structural performance, durability, and scalability in additive manufacturing of concrete.

## Figures and Tables

**Table 1:** Granulometry of each material.

Material	D10 (µm)	D50 (µm)	D90 (µm)
Sand	460	680	996
Limestone filler	1.71	6.8	33.8
Cement	2.27	13.9	44.2

**Table 2:** Technical properties of the glass fiber mesh (as provided by the manufacturer, Würth).

Mesh Type	Mesh Measurements (mm)	Tensile Strength (N/5 cm)	Elongation at Break (%)	Thickness (mm)	Weight (gr/m^2^)
Glass fiber mesh	4.0 × 5.0 (±10%)	2100 (±5%)	4	0.60	160 (±10%)

**Table 3:** Technical properties of the ARMO-mesh 200/200 and 500/500 (as provided by the manufacturer, S&P).

Mesh Type	Mesh Opening (mm)	Modulus of Elasticity (kN/mm^2^)	Tensile Strength (MPa)	Elongation at Break (%)	Thickness of Carbon Fiber (Weight/Density) (mm)	Tensile Strength at Elongation at Break (Width: 1000 mm) (kN)
ARMO-mesh 200/200	16 × 16 (±10%)	≥240/240	≥4400/≥4300	1.8–2.1/1.8	0.044	193/189
ARMO-mesh 500/500	13 × 13 (±10%)	240/250	≥4300/4300	1.8/1.7	0.105	451

**Table 4:** Mortar composition. The values are obtained by weights ratio.

Sand:Cement Ratio	Limestone Filler	Superplasticizer (SP)	Water/Binder
2:1	10% of sand	1% of cement	0.30

**Table 5:** Approximate batch proportions.

Component	Amount per Batch (kg)
Cement (CEM I 42.5R)	20.0
Sand (max 0.5 mm)	40.0
Limestone filler	4.0
Water	7.5
Superplasticizer	0.3
Total	71.8

**Table 6:** Summary of sample types, reinforcement layouts, number of specimens, and loading conditions.

Sample Type	Fabrication	Reinforcement	Number of Samples	Test Type
Control (None)	3D print	None	≥9	Flexural, Compression (cast)
Glass Fiber Mesh	3D print	Glass mesh (Würth)	≥9	Flexural
ARMO 200/200	3D print	Carbon mesh 200/200	≥9	Flexural
ARMO 500/500	3D print	Carbon mesh 500/500	≥9	Flexural
All above	Casting	As above	≥9 per type	Flexural, Compression (ctrl)

**Table 7:** Average results of flexural in the vertical load direction. For ARMO-mesh 500/500, displacement of 10 mm is allowed. Values are reported as mean ± standard deviation (n ≥ 9 per configuration).

Reinforcement Type	Flexural Strength (MPa)		Standard Deviation	
	7 Day	28 Day	7 Day	28 Day
No reinforcement	7.32	12.21	0.18	0.21
Glass fiber mesh 4 × 5 mm	7.68	12.55	0.22	0.36
ARMO-mesh 200/200	19.76	19.93	0.19	0.22
ARMO-mesh 500/500	23.07	24.4	0.11	0.14

**Table 8:** Average flexural strength of beam-like printed specimens under vertical loading at 28 days (MPa). Values are reported as mean ± standard deviation (n ≥ 9 per configuration).

Reinforcement Type	Flexural Strength (MPa)	Standard Deviation
	28 Day	
No reinforcement	4.56	0.38
ARMO-mesh 200/200	8.53	0.98
ARMO-mesh 500/500	16.3	0.31

**Table 9:** Average flexural strength of small printed specimens under horizontal loading at 7 and 28 days (MPa). Values are reported as mean ± standard deviation (n ≥ 9 per configuration).

Reinforcement Type	Flexural Strength (MPa)		Standard Deviation	
	7 Day	28 Day	7 Day	28 Day
No reinforcement	7.4	8.35	0.16	0.18
Glass fiber mesh 4 × 5 mm	8.27	9.95	0.37	0.33
ARMO-mesh 200/200	9.36	12.02	0.24	0.29
ARMO-mesh 500/500	12.72	15.66	0.55	0.41

**Table 10:** Average flexural strength of beam-like printed specimens under horizontal loading at 28 days (MPa). Values are reported as mean ± standard deviation (n ≥ 9 per configuration).

Reinforcement Type	Flexural Strength (MPa)	Standard Deviation
	28 Day	
No reinforcement	2.2	0.12
ARMO-mesh 200/200	4.69	0.16
ARMO-mesh 500/500	6.3	0.34

**Table 11:** Average flexural strength of cast specimens under vertical loading (MPa). Values are reported as mean ± standard deviation (n ≥ 9 per configuration). * For ARMO-mesh 500/500, tests were continued up to 10 mm displacement.

Reinforcement Type	Flexural Strength (MPa)		Standard Deviation	
	7 Day	28 Day	7 Day	28 Day
No reinforcement	7.77	11.52	0.11	0.14
Glass fiber mesh 4 × 5 mm	8.14	12.45	0.21	0.29
ARMO-mesh 200/200	18.87	22.72	0.17	0.32
ARMO-mesh 500/500 *	22.44	27.19	0.33	0.19

**Table 12:** Average results of compression strength of cast specimens under vertical loading. Values are reported as mean ± standard deviation (n ≥ 9 per configuration).

Reinforcement Type	Flexural Strength (MPa)		Standard Deviation	
	7 Day	28 Day	7 Day	28 Day
No reinforcement	44.04	49.78	0.44	0.36

**Table 13:** Average flexural strength of cast specimens under horizontal loading (MPa). Values are reported as mean ± standard deviation (n ≥ 9 per configuration).

Reinforcement Type	Flexural Strength (MPa)		Standard Deviation	
	7 Day	28 Day	7 Day	28 Day
No reinforcement	7.37	11.52	0.14	0.16
Glass fiber mesh 4 × 5 mm	7.6	12.23	0.29	0.18
ARMO-mesh 200/200	12.98	15.44	0.17	0.21
ARMO-mesh 500/500	17.42	19.1	0.23	0.27

**Table 14:** Average compressive strength of unreinforced cast specimens under horizontal loading (MPa). Values are reported as mean ± standard deviation (n ≥ 9 per configuration).

Reinforcement Type	Flexural Strength (MPa)		Standard Deviation	
	7 Day	28 Day	7 Day	28 Day
No reinforcement	46.94	49.78	0.24	0.13

**Table 15:** Comparison with previous studies on reinforcement in 3D-printed concrete.

Study	Year	Reinforcement Type	Key Findings	Novelty/Differences in Current Study
Panda et al. [12]	2017	Short fibers	15–30% increase in flexural strength; anisotropic performance	Uses continuous wide horizontal carbon meshes; achieves up to 100% flexural enhancement; focuses on mesh geometry and porosity effects
Hambach & Volkmer [13]	2017	Fiber-reinforced paste	Modest strength gains with short fibers	Surpasses with porous carbon meshes (ARMO 500/500); indirect quantification of interlayer bonding improvement
Wang et al. [14]	2020	AR-glass textile	~60% improvement in load bearing, mainly ductility	Confirms marginal gains for glass mesh; carbon meshes outperform by up to 70%; systematic horizontal integration during printing
Classen et al. [16]	2020	Additive steel/concrete	Improved flexural capacity with metallic systems	Introduces wide-format non-metallic carbon meshes; corrosion-resistant; up to 100% strength gain over controls
Marchment & Sanjayan [21]	2020	Overlapping steel meshes	Marked improvements in flexural capacity and ductility	Uses non-metallic carbon fiber meshes; higher gains (100%) with wide porous format; horizontal placement focus
Wang et al. [18]	2021	U-nails for interlayer	145% tensile and 220% shear increase at interfaces	Complementary approach; meshes provide continuous bridging for flexural/post-cracking enhancement
Liu et al. [22]	2023	Various 3DPC components	Review of mechanical properties and anisotropy	Provides empirical comparison carbon vs. glass; quantifies size effects and superior performance of ARMO-mesh 500/500
Nan et al. [23]	2025	Various (steel, fibers, meshes)	Reviews challenges and innovations in reinforcement	Addresses key gaps with automated-compatible horizontal carbon meshes; sets new benchmark (100% flexural increase)
Sagyntay et al. [20]	2025	Standard rebar meshes	Automated production with integrated bars	Focuses on non-metallic fiber meshes; lightweight/corrosion-resistant; higher flexural gains vs. rebar-based
Ding et al. [25]	2025	Flexible FRP strips/grids	Bond tests and concept for FRP reinforcement	Complements with wide carbon mesh integration; emphasizes porosity and thickness for superior interlayer stress transfer and 100% flexural improvement

## Data Availability

The original contributions presented in this study are included in the article. Further inquiries can be directed to the corresponding author.

## Abbreviations

The following abbreviations are used in this manuscript:

**Abbreviation/Symbol**	**Description**
OPC	Ordinary Portland Cement, the main binder used in mortar mix
D10, D50, D90	Particle size percentiles (in μm): diameters at which 10%, 50%, and 90% of particles are finer
Elongation at break (%)	Maximum strain (%) the glass fiber mesh can undergo before rupture (manufacturer data)
Thickness (mm) glass fiber mesh	Physical thickness of the glass fiber mesh (as reported in product data)
Weight (gr/m^2^) glass fiber mesh	Mass per unit area of glass fiber mesh
Tensile strength (N/5 cm) glass fiber mesh	Ultimate tensile force per 5 cm width strip for glass mesh (manufacturer’s reported property)
ARMO-mesh 200/200	Product name/code for carbon fiber mesh grade 200/200
ARMO-mesh 500/500	Product name/code for carbon fiber mesh grade 500/500
Modulus of elasticity (kN/mm^2^) carbon fiber mesh	Young’s modulus describing stiffness of carbon fiber mesh
Thickness of carbon fiber (mm)	Physical thickness of the carbon fiber mesh, as reported or measured
Tensile strength at elongation at break (width: 1000 mm) (kN) carbon fiber mesh	Maximum tensile force for a 1000 mm strip of carbon mesh at ultimate strain
AR	Sample designation denoting “As Reinforced” (used for mesh-reinforced specimens)
CS	Compressive strength: maximum compressive load per area, as measured by standard tests
FS	Flexural strength: maximum stress during three-point flexural (MPa)
F	Applied mechanical force (load), used in strength calculations
A	Cross-sectional area subjected to load during mechanical tests
L	Specimen/support span in flexural strength (mm)
B	Width of specimen (mm)
H	Height/thickness of beam specimen (mm)
